# Developing a quality index and an evaluation indicator system for the National Food Safety Standard Framework in China

**DOI:** 10.1057/s41271-026-00620-1

**Published:** 2026-01-21

**Authors:** Hao Ding, Di Wu, Hanyang Lyu, Xin Zhang, Yongxiang Fan, Jing Tian

**Affiliations:** 1 China National Center for Food Safety Risk Assessment, 37 Guangqu Lu, Bldg 2, Chaoyang, Beijing, 100022 People’s Republic of China; 2 Sichuan University, Chengdu, 610041 Sichuan China; 3 Harbin Medical University, Harbin, 150088 Heilongjiang China

**Keywords:** China National Food Safety Standard Framework, Quality index, Evaluation indicator system, Delphi method, Confirmatory factor analyses

## Abstract

International Organization for Standardization and Codex Alimentarius Commission emphasized the importance of evaluating and monitoring food safety. National food safety standard framework (NFSSF)—a generic term for all national food safety standards of China—is an important part of the country’s food control system implemented over ten years. To evaluate NFSSF, the China National Center for Food Safety Risk Assessment developed a quality index with an indicator scale. This study assessed this index with a three-layer evaluation indicator system (NFSSF_eis_) based on three dimensions, namely, scientific nature, relevance, and coordination, using 10 s-layer indicators weighed with the Delphi method. We conducted a pilot survey to test the scale built based on NFSSF_eis_. Confirmatory factor analyses indicated that items in the scale have acceptable reliability and validity. With the development of the NFSSF_eis_, future research is needed to refine this instrument.

**Supplementary Information:**

The online version contains supplementary material available at 10.1057/s41271-026-00620-1.

## Key messages

- We developed an online evaluation indicator system for the National Food Safety Standard Framework (NFSSF) of China based on a Delphi study, and validation by confirmatory factor analysis (CFA).
- We conducted the first-time assessment of the NFSSF and determined the comprehensive score of 8.92, reflecting that the quality of the NFSSF is acceptable.

## Introduction

Food is a paramount necessity of all people, and food safety is essential for public health. To ensure food safety, many international associations and countries have constructed their respective food safety control systems. In general, these food safety control systems contain four main parts: food safety risk analysis, management, communication, and surveillance [1]. For food safety risk management, food safety standards are one of the major management tools and have an important role in food safety control systems [2]. With the implementation of food safety control systems in China, the following questions have been raised by policymakers: How are food safety control systems characterized? Does this control system cover all food risk factors? How do these systems perform? These questions call for the evaluation of food safety control systems.

The International Organization for Standardization (ISO) has emphasized the importance of evaluating the performance of food safety control systems according to the *Food Safety Management Systems*—*Requirements for Organizations in the Food Chain* (ISO22000:2018) [3]. Moreover, the Codex Alimentarius Commission (CAC) has provided the *Principles and Guidelines for Monitoring the Performance of National Food Control Systems* (CXG 91:2017) [4], which aim to support the self-assessment of different national food safety control systems. The CAC guidelines recommend utilizing a performance monitoring framework to monitor food safety control systems. This framework comprises six steps: preparation, defining the evaluating objectives, establishing indicators, creating a monitoring plan, collecting and analyzing data, and reporting and consolidating findings. Inspired by the CAC guidelines, the National Center for Food Safety Risk Assessment (CFSA) established a study group and conducted a project to evaluate the China Food Safety Standard Framework (NFSSF), a generic term for all national food safety standards (and their structure) of China [5].

**Fig. 1:**
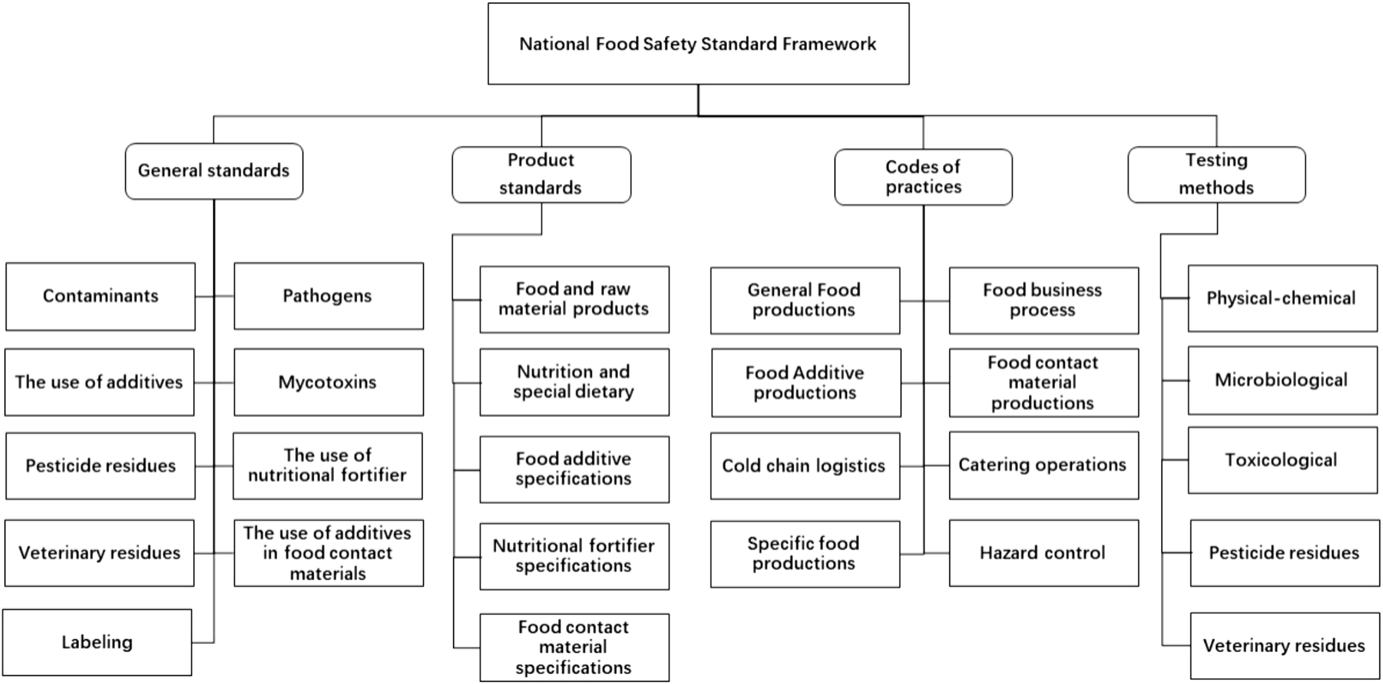
National food safety standard framework of China

The official development of NFSSF started in 2009, when the China Food Safety Law (promulgated in 2009) first mentioned NFSS and the initial framework of NFSS had been designed. To implement the framework, an integration program was completed from 2013 to 2015, and over 5000 existing food standards were reviewed and integrated [6], leading to the establishment of a national-level food safety standard framework. After over 10 years of evolution, the NFSSF has 1478 mandatory standards classified into four categories: (1) general standards; (2) food product standards, food additive and food fortifier specifications, and food contact material standards; (3) code of practice for food production and distribution; and (4) inspections and testing methods (Fig. 1).

To establish indicators for policy evaluation or regulatory impact assessment, many researchers have explored a variety of methodologies. Among these methods are cost-effectiveness analysis (CEA) and cost–benefit analysis (CBA), especially for health and medical practice [7–9]. In 2003, the World Health Organization (WHO) published a guideline for CEAs to identify ways for redirecting resources [10]. However, CEA and CBA are difficult to apply for evaluating food safety policies, especially for NFSSF, because there are too many confounders, and cost and benefit are difficult to measure and quantify [11, 12]. In fact, accurately quantifying the benefits of preventing foodborne illnesses is multifaceted. The benefits may include reduced healthcare costs and productivity losses, and enhanced public health and well-being, which are often difficult to assign precise monetary values.

Given the obstacles that CBAs and CEAs encounter in practice, multi-criteria decision analysis was suggested as an alternative based on different measurement scales [13].

In 2020, a measurement scale was designed for NFSS [11], evaluating the quality of a specific standard [14] or a group of standards [15, 16]. The next step was to recognize standards as a framework and examine whether the design of the framework is scientifically sound, its elements in the framework are necessary and functional, the position of the framework in the entire regulatory system, and the relationship of the framework with other factors in or out of the regulatory system. To better address these issues, CFSA aimed to develop a quality index (QI) with an indicator system and scale to evaluate NFSSF. The evaluation results are to assist food safety officers in decision-making and public communication. This study introduces the development of an evaluation indicator system for NFSSF through the indicator selection using a modified Delphi method and indicator system validation by confirmatory factor analysis (CFA).

## Data and methods

### Survey design

**Fig. 2:**
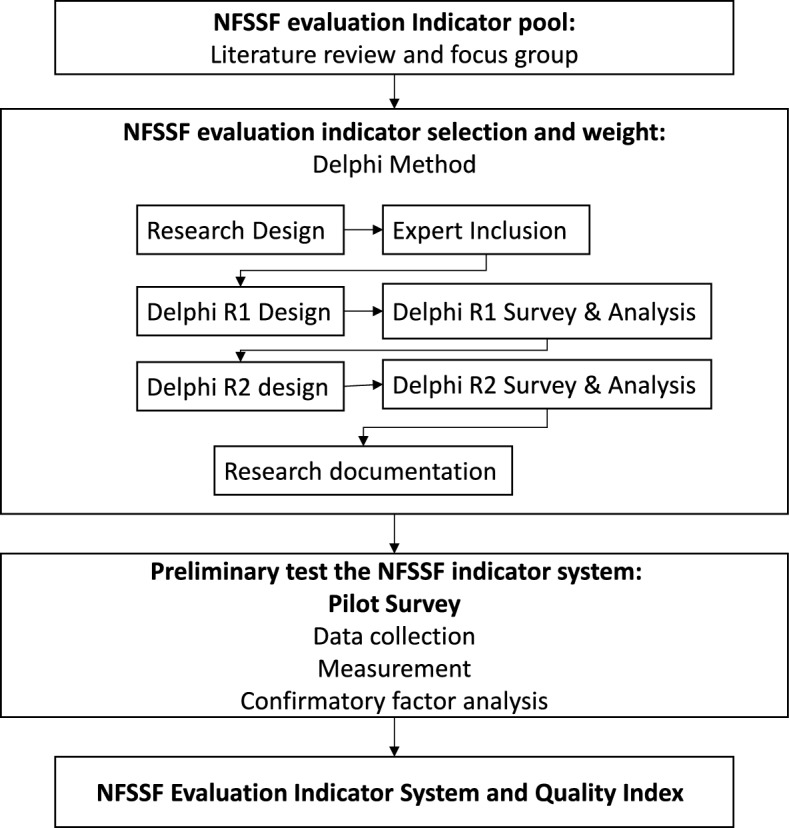
Study procedures

The Delphi method is a popular technique that facilitates decision-making based on structured opinions and comments of experts [17]. The classical Delphi method includes three steps: (1) identify the research subject, specify the research question, establish the rudimentary conceptual model, and design the appropriate questionnaire; (2) identify and select a panel of participating experts; and (3) conduct a survey to collect experts’ opinions, which normally involves two or more rounds [18, 19]. The current study used the Delphi method and followed the classical steps. First, literature review and focus group discussions helped prepare the questionnaire, including the indicator pool and each indicator’s definition. Meanwhile, an expert panel was formed through rigorous selection, and the Delphi survey started thereafter. After the two-round Delphi analysis, experts concurred with the multi-layer evaluation indicator system for NFSSF. Finally, we applied the indicator system and conducted a pilot survey to validate the NFSSF evaluation indicators by CFA. The study procedures (Fig. 2), including questionnaire distribution and comment collection, were implemented and tracked on a web-based platform designed for this study [20].

### Delphi study

CFSA assembled a large expert pool from the national food safety standard review committees and those involved in the life cycles of standards. An expert competency model was designed to decide if the experts’ expertise or working experience matched the aim of this study. We used a 100-point score of the expert competency with four dimensions weighed as: 10—background (an expert’s degree, professional title, position, working years (or age), and the nature of the work); 20—know-how and skill set (working activities, involvement in professional committees, and related academic achievements to judge whether the expert’s knowledge background and structure met the evaluation needs); 60—anchored behavior (expert’s understanding and familiarity with the standard framework to identify whether experts participate in standard’s drafting, review, and application); and 10—engagement (active participation of experts in the survey). We assessed experts’ competency using this model, and those whose comprehensive marks were above 50 were included in the expert panel.

### Data collection and analysis

We designed an electronic questionnaire to include: (1) introduction of the study, (2) basic information on experts, and (3) rudimentary version of the multi-layer evaluation indicators for NFSSF. Based on literature review and focus groups, we drafted a three-layer indicator system to evaluate the quality and effectiveness of NFSSF. The selected experts were asked to weigh all indicators based on their importance and indicators’ contributions to the entire evaluation result using a 10-point Likert-type scale with “1” indicating that the indicator is the least important, “10” is the most important, and “0” meaning the suggestion to delete this indicator. Participants can revise the expression and definition of each indicator and add or delete indicators. According to the suggestions after Round 1, we optimized the wording and expression and specified the explanations of several indicators. Given that two experts replied that they had concerns with “if the NFSSF covers food-related products,” we provided a supplemental instruction to emphasize the composition of NFSSF. Several participants suggested to add a part in the questionnaire to offer rating reasons, through which the study group could obtain the rates and also the reasons behind the scores. During the two-round Delphi study, if the result suggests that all third-layer indicators under a second-layer indicator were recommended to be deleted, then the second-layer indicator will be deleted, and vice versa.

The online platform provided statistics with an embedded analysis program and calculated several estimates (see Table 1): the mean (*M*_j_), weighted mean (*X*_j_), full mark rate (*K*_j_) to represent the concentration degree of expert opinions on the importance of the indicators, and coefficient of variation (*V*_j_) to measure the dispersion of expert opinions (the lower the coefficient of variation, the higher the degree of coordination of experts) [21, 22]. We used the weighted mean to calculate the normalized weights (*W*_j_) of indicators. The indicator weight represents a quantitative distribution of importance in the overall evaluation system [23]. We also calculated the Kendall’s coordination coefficient (w), an indicator of the credibility of the consultation results ranging between 0 and 1, to reflect the consistency of different experts’ opinions and conducted the dominance test of the coordination coefficient with *P* < 0.05. We assumed the coordination coefficient to be significant, and experts’ opinions are reliable for non-accidental coordination [24, 25]. Indicator screening was based on the appraisal of importance [11]: the indicator was excluded if its weighted mean of importance fell below 7 and V_j_ below 0.25; if its weighted mean is above 7 and V_j_ is below 0.25, then the item would be retained. The indicator selection followed the same principle in the two rounds.

**Table 1:** The statistical indexes

Index	Equation
Willingness index of participants	(Response rate) = $\frac{\text{Number of feedback}}{\text{Number of distribution }}\times 100\mathrm{\%}$
Mean (${M}_{j}$)	${M}_{j}=\frac{1}{{m}_{j}}\sum_{i=1}^{m}{C}_{ij}$(${m}_{j}$: number of experts; ${C}_{ij}$: rating value of expert $i$ on indicator $j$)
Weighted mean (${\overline{{\boldsymbol{X}}} }_{{\boldsymbol{j}}}$)	${\overline{X} }_{j}=\frac{\sum_{i=1}^{m}{{W}_{i}*C}_{ij}}{\sum_{i=1}^{m}{W}_{i}}$ (${W}_{i}$: weight value of expert $i$ or competency score of expert $i$)
Full mark rate (${K}_{j}$)	${K}_{j}=\frac{{m}_{j}{\prime}}{{m}_{j}}$(${m}_{j}{\prime}$: number of expert rating “10” on indicator $j$)
Coefficient of variation (${V}_{j}$)	${V}_{j}=\frac{{\sigma }_{j}}{\overline{{\mathrm{x} }_{\mathrm{j}}}}$(${\sigma }_{j}$: standard deviation for indicator $j$)
Coordination coefficient ($\mathrm{w}$)	$\mathrm{w}=\frac{12\sum_{j=1}^{k}{\mathrm{R}}_{\mathrm{j}}^{2}-3{b}^{2}k{(k+1)}^{2}}{{\mathrm{b}}^{2}\mathrm{k}\left({\mathrm{k}}^{2}-1\right)-\mathrm{b}\sum \left({\mathrm{t}}^{3}-\mathrm{t}\right)}$(${R}_{j}$: sum of ranks assigned to indicator $j$; T_j_: rank sum of indicator $j$; $\overline{T}$: mean of the rank sum of indicator $j$; k: number of indicators; b: number of experts; t: number of indicators with the same $\overline{T}$)
Weight (${W}_{j}$)	${W}_{j}$=$\frac{{\overline{{\boldsymbol{X}}} }_{{\boldsymbol{p}}{\boldsymbol{j}}}}{\sum_{p=1}^{n}{\overline{{\boldsymbol{X}}} }_{{\boldsymbol{p}}{\boldsymbol{j}}}}$($j$ ∈ [1,k]. ${\overline{{\boldsymbol{X}}} }_{{\boldsymbol{p}}{\boldsymbol{j}}}$: ${\overline{{\boldsymbol{X}}} }_{{\boldsymbol{j}}}$ under its upper-layer indictor p; n: number of second- third-layer indicators under the same upper-layer indicators)

### Survey evaluation

Fifty-one qualified experts participated in the evaluation survey. Questionnaires were distributed through a web-based platform. Experts received email or text message notifications on the start and progress of each round of evaluation. The willingness of the participants represented the response rate. In general, a response rate is acceptable if it is over 70% [26]. The indicator system established by the Delphi study was applied to conduct the initial assessment of NFSSF. The weighted mean (*X*_j_) of the experts’ rating values was regarded as the final scores of the third-layer items. The assessment results of the quality index (QI) and first- and second-layer indicators were calculated as the sum of products of the weighted mean and indicator weights (*X*_j *_W_j_).

We assessed reliability through internal consistency with Cronbach’s alpha estimates based on covariance and number of items [27]. In this study, with Cronbach’s alpha coefficient of 0.7 or above, the internal consistency of the scale was considered satisfactory [28]. To assess validity and the extent to which the underlying model was fit to the evaluation survey data, we performed CFA. We tested the model fit based on absolute fit [29], and the ratio of Chi-square and degrees of freedom (CMIN/df), and standardized root mean square residual (SRMR) to represent absolute fit. For CMIN/df, a value of < 3 was considered widely acceptable and < 5 as debatable [30]; or SRMR, a value close to 0.08 and below was considered acceptable [31, 32].

Data were analyzed using SPSS 19.0 for the descriptive statistics. The Lavaan package in R was used for CFA [33].

## Results

### Delphi study

After the assessment by the expert competence model, 51 experts qualified and were invited to participate in this Delphi study. Expert competence scores ranged from 51 to 89; only experts with scores over 50 had been selected. These scores were regarded as weights of the participants (*W*_*i*_), revealing the experts’ familiarity and authority on this topic. About 75% of the participants hold master’s or doctoral degrees majoring in food science, medicine, chemistry, microbiology, public health, nutrition, or law. Over 60% of experts held senior titles, and over 94% of the experts worked in closely related work units, including the National Health Committee, Center for Disease Control and Prevention, Customs, State Administration for Market Regulation, Ministry of Agriculture and Rural Affairs, CFSA, and food industry associations, covering the standard lifecycle from drafting to executing. We also invited three qualified professors from universities to provide their opinions from an academic perspective. Most of the included experts had participated in drafting, reviewing, or applying NFSS, and several of them are principal drafters or directors of the National Food Safety Standards Review Committee. The response rates were 100% for both rounds, representing the high willingness of participation of the invited experts.

**Round I** None of the indicators met the exclusion criteria in Round 1. All indicators at each layer gained a high weighted mean of over 8 on importance. Expert opinions were highly unanimous, as revealed by the low coefficient of variations, ranging from 0.05 to 0.21. The importance of the three dimensions (relevance, scientific nature, and coordination) was rated as 9.86, 9.79, and 9.52, respectively, with coordination having the lowest importance score and the importance score of relevance being the highest of the three dimensions and among all indicators. Relevance had the highest full mark rate of 92%, while its third-layer item (“The NFSSF is in line with the development direction of food and food-related industries and encourages innovation”) received the lowest mark at 45%. The participants actively commented on the multi-layer indicators and their explanations. A total of 19 written comments were received in Round 1 from different experts. Moreover, there were 11 comments on the wording and expression of indicators. Three new indicators (“The standards and regulations in the NFSSF realize risk-based classified and hierarchical management”; “The NFSSF provides guidance for market regulators to improve their capacities”; and “The NFSSF helps control safety risk at a reasonable cost”) were added to 40 indicators to optimize the evaluation system according to expert comments for the Round 2 assessment.

**Round II** A total of 43 indicators were included for the second-round review. The outcome was consistent with the one in Round 1. That is, all indicators received above 7 (7.75 to 9.76) on importance, while their coefficients of variation were below 0.25. A consensus on the experts’ opinions was reached again in Round 2. The order of the items by their weighted means on importance is as follows: scientific nature (9.69) replaced relevance (9.60), remaining at the top among the three principal criteria. Moreover, the second-layer indicator (“Health promotion for consumers”) under relevance had the highest importance score of 9.76. In contrast, the indicator “The NFSSF is in harmonization with international standards” did not obtain a full mark, and the weighted mean of an indicator (“The NFSSF roughly matches with the Codex standard system and the food safety standard system of other developed countries in the world”) has the lowest score of 7.75. The coordination coefficients of the two rounds were 0.127 (Round 1) and 0.381 (Round 2) (*P* < 0.05), suggesting that expert opinions were highly reliable without accidental coordination. Lastly, all indicators met the inclusion criteria, and the three-layer indicator evaluation system was established for NFSSF, consisting of 43 indicators, with 3 (first layer), 10 (second layer), and 30 (third layer). The weights of the three dimensions are 0.341 (scientific nature), 0.338 (relevance), and 0.321 (coordination). The indicator weights of NFSSF_eis_ and the final definitions of the third-layer indicators are presented in the Supplementary Material.

### Evaluation survey

**Fig. 3:**
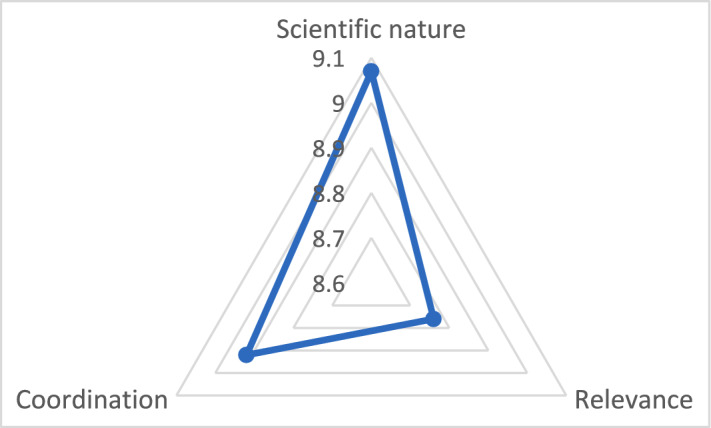
Final scores of the three dimensions (i.e., scientific nature, relevance, and coordination of the national food safety standard framework)

**Fig. 4:**
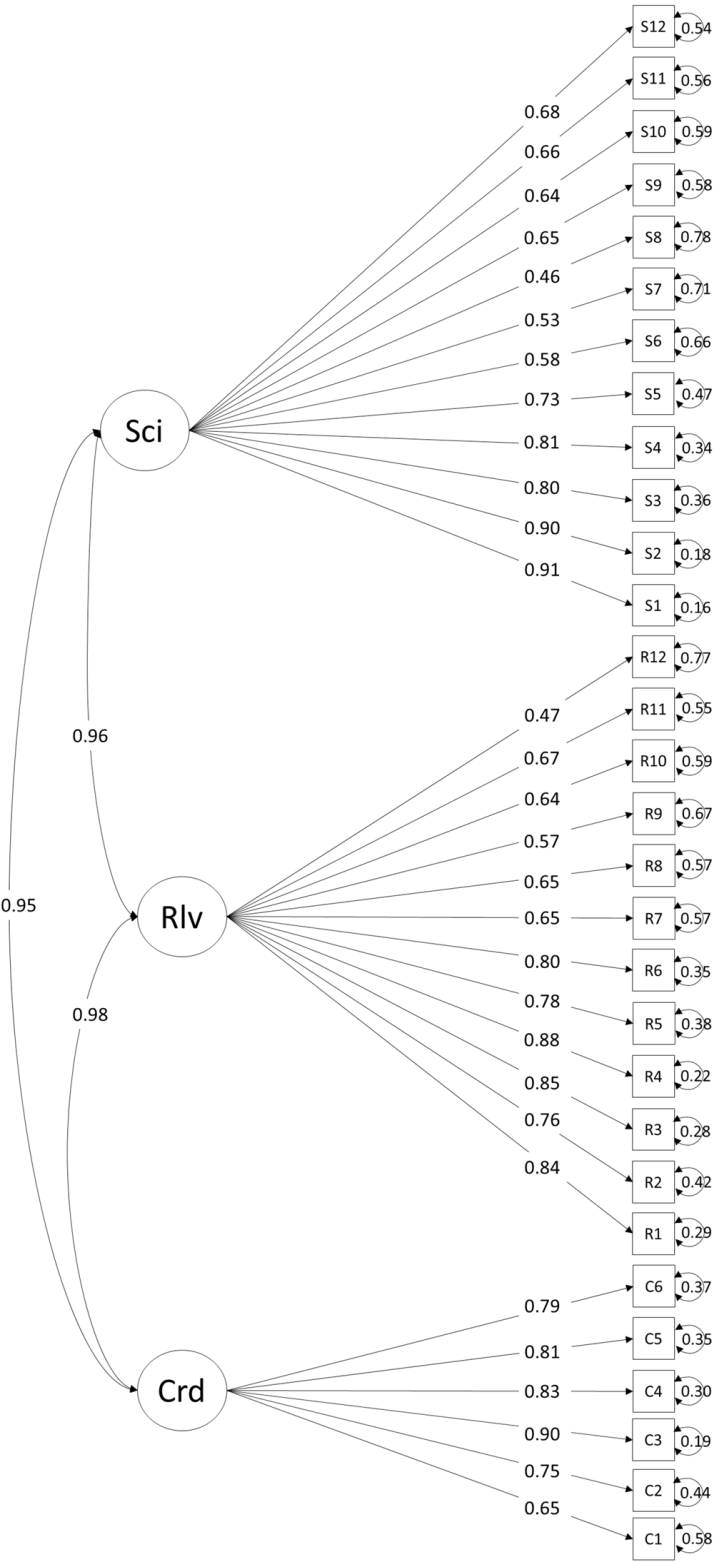
Three-dimensional confirmatory factor analysis model. Sci scientific nature, Rlv relevance, Crd coordination

Overall, the response rates were 100%; QI of NFSSF was 8.92, while a full mark was 10. The scores of the three main dimensions were high (scientific nature—9.07; coordination—8.92; and relevance—8.76), as shown in Fig. 3. All items and third-layer indicators were rated above 8, and most of their scores were above 8.5. This high score of QI and all indicators was consistent with the generally good comments on NFSSF from the experts [34]. QI and indicators showed the experts’ comments in a quantified manner. The Cronbach’s alpha coefficient is 0.968, exceeding Nunnally’s criteria of 0.7. The reliability of the evaluation scale or QI was found to be satisfactory, representing a high correlation between items. The scale was analyzed for construct validity using CFA. The independent items showed acceptable standardized loadings to the three dimensions (ranging between 0.46 and 0.91) (Fig. 4). Hence, the scale was considered to have good construct validity. CFA also showed that CMIN/df = 2.281 and SRMR = 0.082. The values of CMIN /df and SRMR were acceptable.

## Discussion

In this study, we aimed to develop a quality index to evaluate the NFSS framework and facilitate the decision-making of food safety policy in China. During the Delphi study, the experts reached consensus on the indicators and the structure of the evaluation system; the pilot survey validated scientific nature, relevance, and coordination as the three main dimensions to reflect the quality of NFSSF, and the validity and reliability of the scale were considered acceptable.

The three newly added indicators after the Delphi survey refined the indicator system. This update conforms with the research tendency on food safety risk ranking in China [35, 36]. Food safety risk ranking during the development of the national food safety standards contributes to the reasonable allocation of the limited resources of food safety control, improvement of food safety control efficiency, and lightened supervision load [37–39]. Another newly added item reflected the similar principle of cost control, consistent with the current economic opinions in food safety control [40–42]. Finally, the item related to market regulation is critical for grassroots actions [34, 43].

The indicators focusing on health protection for consumers obtained the highest importance score, as the vital goal of NFSSF and all national food safety standards [5, 44]. Physical and chemical pollutants, microbiological contaminants, veterinary drugs, pesticides, and food additives are key issues that NFSSF discusses and controls [45, 46]. By contrast, the third-layer item related to the national nutrition level obtained the lowest score on importance. One expert merely gave a score of 6, commenting that “The NFSSF’s main purpose is protecting safety, so the weight value of the standards for nutrition limits and labels should not be overly reflected.” Note that among all indicators, the weighted mean of an indicator reflecting the match between NFSSF and the Codex standard system and the food safety standard system of other developed countries in the world was the lowest (7.75) in Round 2. The experts suggested that policies or standards should be relevant to national circumstances. Reliability (internal consistency) and the structure validity of the scale were acceptable. The two absolute fit indices of the three-dimension model were acceptable, suggesting that the model generally fit the data. Absolute fit does not consider the fit of the chosen model compared with another model, and it is based only on how well the model is able to recreate the correlation matrix of the data [29].

### Study limitations

For the pilot survey, the sample size of 51 qualified experts was inadequate. In general, the sample size used to validate a model or scale should be no less than five times the number of the items/indicators in the scale. Ideally, the sample should be over 150 [29]. Given the special nature of our evaluation objective, the number of experts qualified to conduct the evaluation is limited. Fit statistics used in CFA can be classified into three categories: absolute fit, parsimony correction, and comparative fit [29]. The current study only selected the indexes under absolute fit because we just planned to generally validate the model in this step. Typically, parsimony correction and comparative fit are used to select the ideal model from other model candidates. To further refine the model, the software could suggest several model modifications, although the suggested modifications were not compatible with the theoretical concept of NFSSF_eis_ constructed by the Delphi study. Therefore, the model modifications were not considered. To expand this exploratory study, in future research we could increase the sample size and further validate and refine the evaluation indicator system of NFSSF as the digitization of the indicator system will be available, and the regular and dynamic evaluation of the standard framework will be carried out.

## Conclusions

In this study, we designed a three-layer indicator system, and each indicator was weighed to evaluate NFSSF from three dimensions, namely, scientific nature, relevance, and coordination, using the two-round Delphi method. This version of NFSSF_eis_ showed acceptable construct validity and reliability, making it a potential tool for assessing the quality of NFSSF. Lastly, further research is needed to refine the instrument. We propose this indicator system not as a definitive tool, but as an initial attempt to operationalize the evaluation principles encouraged by CAC and ISO. For the international community, we hope our work may serve as a starting point for dialogue. It offers a case study from China that might inspire similar exploratory efforts in other national contexts, contributing to the global conversation on how to effectively assess and strengthen food safety governance. We invite further refinement and adaptation of this framework by researchers and policymakers worldwide.

## Supplementary Information

Below is the link to the electronic supplementary material.

Supplementary file1 (DOCX 59 KB)

## Data Availability

Data available on request from the corresponding author.
