# Supplementary material for: Developing a quality index and an evaluation indicator system for the National Food Safety Standard Framework in China
Source: J Public Health Policy. 2026 Jan 21;47(1):99–112. doi: 10.1057/s41271-026-00620-1 (PMC13008756; doi:10.1057/s41271-026-00620-1)
Supplement: Supplementary file 1 — Supplementary file1 (DOCX 59 KB) [file 41271_2026_620_MOESM1_ESM.docx]

**Supplementary material**

S1 Weights of the indicators in the evaluation system of the national food safety framework in China

| First-layer indicator | | Weight | Second-layer indicator | | Weight | | Third-layer indicator | | Weight | |
| --- | --- | --- | --- | --- | --- | --- | --- | --- | --- | --- |
| 1 | Scientific nature | 0.3414 | 1.1 | Scientific nature of the structure | | 0.3339 | 1.1.1 | The NFSSF covers the whole food chain from farmland to tables. | | 0.1943 |
|  |  |  |  |  | |  | 1.1.2 | The NFSSF covers all food categories. | | 0.1897 |
|  |  |  |  |  | |  | 1.1.3 | The NFSSF implements necessary controls of the identified hazards in food chains. | | 0.2150 |
|  |  |  |  |  | |  | 1.1.4 | The NFSSF has a clear position in the food safety control system | | 0.1936 |
|  |  |  |  |  | |  | 1.1.5 | The standards and regulations in the NFSSF realize risk-based classified and hierarchical management. | | 0.2074 |
|  |  |  | 1.2 | Scientific nature of the process and mechanism | | 0.3343 | 1.2.1 | The standards in the NFSSF conduct a complete formulation and revision procedure. | | 0.3520 |
|  |  |  |  |  |  |  | 1.2.2 | Stakeholders participate in the development of the NFSSF. | | 0.3226 |
|  |  |  |  |  |  |  | 1.2.3 | A mechanism for re-evaluation and improvement of the NFSSF has been established. | | 0.3254 |
|  |  |  | 1.3 | Scientific nature of the internal structure | | 0.3318 | 1.3.1 | The NFSSF sets up a clear food classification system. | | 0.2465 |
|  |  |  |  |  |  |  | 1.3.2 | All parts of the NFSSF are necessary. | | 0.2411 |
|  |  |  |  |  |  |  | 1.3.3 | All parts of the NFSSF are closely connected. | | 0.2573 |
|  |  |  |  |  |  |  | 1.3.4 | The internal structure of the NFSSF is integral. | | 0.2551 |
| 2 | Relevance | 0.3381 | 2.1 | Health protection for consumers | | 0.2716 | 2.1.1 | The NFSSF contributes to food safety for consumers. | | 0.3560 |
|  |  |  |  |  |  |  | 2.1.2 | The NFSSF promotes the national nutrition level. | | 0.3091 |
|  |  |  |  |  |  |  | 2.1.3 | The NFSSF reflects the care for vulnerable populations in terms of food safety and health protection. | | 0.3349 |
|  |  |  | 2.2 | Facilitation of market supervision | | 0.2583 | 2.2.1 | The NFSSF provides guidance for market regulators to improve their capacities. | | 0.2517 |
|  |  |  |  |  | |  | 2.2.2 | The standards in the NFSSF provide a basis for market regulators to regulate final products. | | 0.2487 |
|  |  |  |  |  | |  | 2.2.3 | The standards in the NFSSF provide a basis for market regulators to regulate manufacture processes. | | 0.2581 |
|  |  |  |  |  | |  | 2.2.4 | The standards in the NFSSF provide market regulators with testing methods as a reference. | | 0.2415 |
|  |  |  | 2.3 | Promotion of industry development | | 0.2364 | 2.3.1 | The NFSSF improves the production levels across food and food-related industries. | | 0.2489 |
|  |  |  |  |  |  |  | 2.3.2 | The NFSSF regulates the market and avoid unfair competition. | | 0.2417 |
|  |  |  |  |  |  |  | 2.3.3 | The NFSSF is in line with the development direction of food & food-related industries and encourages innovation. | | 0.2417 |
|  |  |  |  |  | |  | 2.3.4 | The NFSSF helps control food safety risk at a reasonable cost. | | 0.2677 |
|  |  |  | 2.4 | Meeting social needs | | 0.2337 | 2.4.1 | The NFSSF meets the specific social needs. | | 1.0000 |
| 3 | Coordination | 0.3205 | 3.1 | The NFSSF is in line with the superior policies. | | 0.3635 | 3.1.1 | The NFSSF is line with the laws and regulations, such as the Food Safety Law. | | 0.5068 |
|  |  |  |  |  |  |  | 3.1.2 | The NFSSF is in line with the current national policies and development directions. | | 0.4932 |
|  |  |  | 3.2 | The NFSSF is in line with other standards and departmental regulations. | | 0.3263 | 3.2.1 | The NFSSF is closely connected with the edible agricultural product management system. | | 0.3331 |
|  |  |  |  |  |  |  | 3.2.2 | The NFSSF is complementary to the management of food safety by other relevant government departments. | | 0.3378 |
|  |  |  |  |  |  |  | 3.2.3 | The NFSSF has a clear relationship with the food quality standard system. | | 0.3291 |
|  |  |  | 3.3 | The NFSSF is in harmonization with the international standards | | 0.3102 | 3.3.1 | The NFSSF roughly matches with the Codex standard system and the food safety standard system of other developed countries in the world. | | 1.0000 |

S2 Appraisal of importance and indicator inclusion of the two-round Delphi survey.

| Num | Indicators | Round 1 | | | | Round 2 | | | | |
| --- | --- | --- | --- | --- | --- | --- | --- | --- | --- | --- |
|  |  | Mean (Mj) | Weighted Mean  ($\bar{X}_{j}$) | Full Mark Rate (Kj%) | Coefficient of Variation (Vj) | Mean (Mj) | Weighted Mean  ($\bar{X}_{j}$) | Full Mark Rate (Kj%) | Coefficient of Variation (Vj) | Decision |
| 1 | Scientific nature | 9.80 | 9.79 | 90 | 0.073 | 9.71 | 9.69 | 82 | 0.080 | K^a^ |
| 1.1 | Scientific nature of the structure | 9.65 | 9.63 | 78 | 0.079 | 9.47 | 9.46 | 69 | 0.121 | K |
| 1.1.1 | NFSSF covers the entire food chain from farmland to tables. | 9.47 | 9.44 | 71 | 0.097 | 8.61 | 8.60 | 20 | 0.154 | K |
| 1.1.2 | NFSSF covers all food categories. | 9.25 | 9.23 | 67 | 0.137 | 8.41 | 8.40 | 6 | 0.131 | K |
| 1.1.3 | NFSSF implements necessary controls of the identified hazards in food chains. | 9.65 | 9.63 | 80 | 0.087 | 9.53 | 9.52 | 75 | 0.122 | K |
| 1.1.4 | NFSSF has a clear position in the food safety control system. | 9.35 | 9.34 | 65 | 0.108 | 8.59 | 8.57 | 24 | 0.206 | K |
| 1.1.5 | Standards and regulations in NFSSF realize risk-based classified and hierarchical management. ^b^ | / | / | / | / | 9.22 | 9.18 | 57 | 0.135 | K |
| 1.2 | Scientific nature of the process and mechanism. | 9.71 | 9.70 | 80 | 0.069 | 9.49 | 9.47 | 67 | 0.115 | K |
| 1.2.1 | Standards in NFSSF conduct a complete formulation and revision procedure. | 9.63 | 9.62 | 76 | 0.077 | 9.47 | 9.45 | 69 | 0.107 | K |
| 1.2.2 | Stakeholders participate in the development of NFSSF. | 9.45 | 9.44 | 67 | 0.090 | 8.69 | 8.66 | 20 | 0.140 | K |
| 1.2.3 | A mechanism for re-evaluation and improvement of NFSSF has been established. | 9.27 | 9.23 | 63 | 0.128 | 8.75 | 8.73 | 20 | 0.137 | K |
| 1.3 | Scientific nature of the internal structure | 9.63 | 9.63 | 80 | 0.092 | 9.39 | 9.40 | 61 | 0.106 | K |
| 1.3.1 | NFSSF sets up a clear food classification system. | 9.00 | 8.97 | 57 | 0.159 | 8.59 | 8.57 | 24 | 0.165 | K |
| 1.3.2 | All parts of NFSSF are necessary. | 9.27 | 9.24 | 65 | 0.145 | 8.39 | 8.39 | 12 | 0.198 | K |
| 1.3.3 | All parts of NFSSF are closely connected. | 9.49 | 9.47 | 73 | 0.109 | 8.94 | 8.95 | 29 | 0.126 | K |
| 1.3.4 | Internal structure of NFSSF is integral. | 9.16 | 9.13 | 69 | 0.200 | 8.86 | 8.87 | 20 | 0.107 | K |
| 2 | Relevance | 9.86 | 9.86 | 92 | 0.049 | 9.61 | 9.60 | 84 | 0.124 | K |
| 2.1 | Health protection for consumers | 9.82 | 9.82 | 90 | 0.056 | 9.76 | 9.76 | 84 | 0.066 | K |
| 2.1.1 | NFSSF contributes to food safety for consumers. | 9.82 | 9.82 | 86 | 0.048 | 9.55 | 9.55 | 75 | 0.100 | K |
| 2.1.2 | NFSSF promotes the national nutrition level. | 8.84 | 8.80 | 55 | 0.177 | 8.31 | 8.29 | 8 | 0.144 | K |
| 2.1.3 | NFSSF reflects the care for vulnerable populations in terms of food safety and health protection. | 9.39 | 9.38 | 69 | 0.099 | 8.96 | 8.98 | 29 | 0.115 | K |
| 2.2 | Facilitation of market supervision | 9.61 | 9.56 | 84 | 0.105 | 9.29 | 9.28 | 61 | 0.119 | K |
| 2.2.1 | NFSSF provides guidance for market regulators to improve their capacities. ^b^ | / | / | / | / | 8.73 | 8.65 | 37 | 0.159 | K |
| 2.2.2 | Standards in NFSSF provide a basis for market regulators to regulate final products. | 9.33 | 9.28 | 71 | 0.127 | 8.59 | 8.55 | 16 | 0.135 | K |
| 2.2.3 | Standards in NFSSF provide a basis for market regulators to regulate manufacturing processes. | 9.22 | 9.17 | 65 | 0.138 | 8.88 | 8.87 | 20 | 0.106 | K |
| 2.2.4 | Standards in NFSSF  provide market regulators with testing methods as a reference. | 8.88 | 8.79 | 55 | 0.169 | 8.35 | 8.30 | 10 | 0.153 | K |
| 2.3 | Promotion of industry development | 9.41 | 9.36 | 73 | 0.123 | 8.49 | 8.49 | 10 | 0.141 | K |
| 2.3.1 | NFSSF improves the production levels across food and food-related industries. | 9.04 | 8.99 | 53 | 0.144 | 8.39 | 8.35 | 12 | 0.155 | K |
| 2.3.2 | NFSSF regulates the market and avoid unfair competition. | 8.76 | 8.71 | 49 | 0.162 | 8.16 | 8.11 | 4 | 0.150 | K |
| 2.3.3 | NFSSF is consistent with the development direction of food and food-related industries and encourages innovation. | 8.65 | 8.60 | 45 | 0.183 | 8.16 | 8.11 | 12 | 0.192 | K |
| 2.3.4 | NFSSF helps control food safety risk at a reasonable cost. ^b^ | / | / | / | / | 9.04 | 8.98 | 49 | 0.131 | K |
| 2.4 | Meeting social needs | 9.33 | 9.26 | 78 | 0.189 | 8.43 | 8.40 | 6 | 0.151 | K |
| 2.4.1 | NFSSF meets the specific social needs. | 8.86 | 8.78 | 51 | 0.205 | 8.37 | 8.35 | 8 | 0.157 | K |
| 3 | Coordination | 9.55 | 9.52 | 78 | 0.108 | 9.14 | 9.10 | 59 | 0.185 | K |
| 3.1 | NFSSF is consistent with superior policies. | 9.71 | 9.68 | 86 | 0.090 | 9.31 | 9.30 | 73 | 0.194 | K |
| 3.1.1 | NFSSF is consistent with laws and regulations, such as the Food Safety Law. | 9.78 | 9.77 | 92 | 0.104 | 9.47 | 9.45 | 80 | 0.174 | K |
| 3.1.2 | NFSSF is consistent with current national policies and development directions. | 9.63 | 9.62 | 78 | 0.113 | 9.22 | 9.20 | 71 | 0.201 | K |
| 3.2 | NFSSF is consistent with other standards and departmental regulations. | 9.47 | 9.46 | 76 | 0.119 | 8.37 | 8.35 | 6 | 0.188 | K |
| 3.2.1 | NFSSF is closely connected with the edible agricultural product management system. | 9.22 | 9.20 | 65 | 0.157 | 8.37 | 8.36 | 16 | 0.199 | K |
| 3.2.2 | NFSSF is complementary to the management of food safety by other relevant government departments. | 9.22 | 9.19 | 63 | 0.148 | 8.49 | 8.47 | 22 | 0.187 | K |
| 3.2.3 | NFSSF has a clear relationship with the food quality standard system. | 8.76 | 8.71 | 51 | 0.196 | 8.29 | 8.25 | 16 | 0.206 | K |
| 3.3 | NFSSF is in harmony with international standards. | 9.37 | 9.36 | 69 | 0.116 | 7.96 | 7.94 | 0 | 0.199 | K |
| 3.3.1 | NFSSF relatively matches with the Codex and food safety standard systems of other developed countries. | 9.12 | 9.11 | 63 | 0.162 | 7.78 | 7.75 | 2 | 0.247 | K |

^a^ K: Keep, the indicator was included into the evaluation system.

^b^ It was a newly added indicator after Round 1.

S3 The definitions of third-layer Indicators

| Num | Indicator | Indicator description |
| --- | --- | --- |
| 1.1.1 | The NFSSF covers the whole food chain from farmland to tables. | The NFSSF covers the whole food chain from farm to fork, which means it covers all steps from primary production to consumption inclusive of the production, processing, storage, transportation and distribution of food and raw materials. |
| 1.1.2 | The NFSSF covers all food categories. | The NFSSF covers all food categories, including fruits, vegetables, edible fungi, cereals, legumes, algae, and their products, nuts and seeds, meat and meat products, fish and aquatic products, dairy and dairy products, eggs and eggs products, oils and their products, condiments, beverages, alcohol beverage, sugar and starch sugar, starch and starch products, baked goods, cocoa products, chocolate and its products, candy, frozen drinks, special dietary foods, etc. |
| 1.1.3 | The NFSSF implements necessary controls of the identified hazards in food chains. | The NFSSF has covered the “limits (MLs or MRLs) of pathogenic microorganisms, pesticide residues, veterinary drug residues, biological toxins, contaminants (heavy metals, etc.), and other substances hazardous to human health in food, food additives, food-related products” mentioned in Article 26 of the China Food Safety Law. According to the results of risk assessment, the necessary control measures have been taken to ensure food safety fundamentally. |
| 1.1.4 | The NFSSF has a clear position in the food safety control system. | In the national food safety management system, the status and role of national food safety standards are clear and definite, relative to other management measures, such as recommended standards, standards developed by group, industry, or private sector, etc. |
| 1.1.5 | The standards and regulations in the NFSSF realize risk-based classified and hierarchical management. | Based on risk analysis and classifications, scientific and accurate management has been achieved by setting different groups of standards such as general standards, product standards, code of practice for food production and distribution, and setting different categories of regulations or indicators and limits in the standards. |
| 1.2.1 | The standards in the NFSSF conduct a complete formulation and revision procedure. | A procedure for formulating and revising the national food safety standards has been established, including project plan, drafting, public consultation, review, approval and issuing, follow-up evaluation, revision, and re-evaluation. |
| 1.2.2 | Stakeholders participate in the development of the NFSSF. | The opinions and suggestions from stakeholders, such as government departments, industry organizations, food companies, university & scientific research institutions, and consumers, have been fully considered during the construction of the national food safety standard framework. |
| 1.2.3 | A mechanism for re-evaluation and improvement of the NFSSF has been established. | The re-evaluation methods and mechanism of the NFSSF have been established; the periodic evaluation and effectiveness evaluation of the NFSSF have been carried out, and suggestions for improving the NFSSF have been put forward according to the evaluation results. |
| 1.3 | Scientific nature of internal structure |  |
| 1.3.1 | The NFSSF sets up a clear food classification system. | A clear food classification system has been established in the NFSSF as well as in the general standards, while a coding system has also been attached. The principles of food classification are consistent, and the settings are reasonable. |
| 1.3.2 | All parts of the NFSSF are necessary. | The NFSSF consists of the general standards, food product standards, food additive and food fortifier specifications and food contact material standards, code of practice for food production and distribution and testing method standards. Such a structure is reasonable, and each part is essential. |
| 1.3.3 | All parts of the NFSSF are closely connected. | In the NFSSF, the general standards, product standards, code of practice for food production and testing method standards perform their respective functions, and their principles are consistent; there is no overlap, repetition, or contradiction between the standards. Unless special circumstances, the content of the product standard involving the general standard shall refer to the general standard; the code of practice for food production and distribution, and inspection methods shall match with the general standard and the product standard. Various standards are coordinated and complementary to each other. |
| 1.3.4 | The internal structure of the NFSSF is integral. | In the NFSSF, the general standards, food product standards, food additive and food fortifier specifications and food contact material standards, code of practice for food production and distribution and testing method standards have been set up, and such a structure can meet the needs of risk management. |
| 2.1.1 | The NFSSF contributes to food safety for consumers. | The current NFSSF can effectively guarantee food safety and protect wellbeing of consumers. |
| 2.1.2 | The NFSSF promotes the national nutrition level. | The regulations regarding nutrition labeling in the NFSSF raise citizens' awareness of food nutrition and facilitate to healthy diets. The dosage and specification requirements on nutritional fortifiers help improve the national nutrition level and promote people’s health. |
| 2.1.3 | The NFSSF reflects the care for vulnerable populations in terms of food safety and health protection. | The NFSSF meets the food safety and health protection needs of vulnerable population such as infants, pregnant women, and the elderly. |
| 2.2.1 | The NFSSF provides guidance for market regulators to improve their capacities. | When some safety specifications or prescribed behavioral requirements are difficult to understand or execute, corresponding guidance are available to help improve the competitive edge of market regulators. |
| 2.2.2 | The standards in the NFSSF provide a basis for market regulators to regulate final products. | The general standards and product standards in the NFSSF provide a basis for [market surveillance authorities](https://context.reverso.net/%E7%BF%BB%E8%AF%91/%E8%8B%B1%E8%AF%AD-%E4%B8%AD%E6%96%87/market+surveillance+authorities) to supervise final products. |
| 2.2.3 | The standards in the NFSSF provide a basis for market regulators to regulate manufacture processes. | The code of practice for food production and distribution in the NFSSF provides a basis for the [market surveillance authorities](https://context.reverso.net/%E7%BF%BB%E8%AF%91/%E8%8B%B1%E8%AF%AD-%E4%B8%AD%E6%96%87/market+surveillance+authorities) to supervise the production and processing process of food enterprises. |
| 2.2.4 | The standards in the NFSSF  provide market regulators with testing methods as a reference. | The testing method standards in the NFSSF provide operation methods and judgment basis for analyses and testing of food and its components for [market surveillance authorities](https://context.reverso.net/%E7%BF%BB%E8%AF%91/%E8%8B%B1%E8%AF%AD-%E4%B8%AD%E6%96%87/market+surveillance+authorities). |
| 2.3.1 | The NFSSF improves the production levels across food and food-related industries. | The regulations and requirements in the NFSSF set the bottom line of food safety, regulate the production, processing and operation of small and medium-sized enterprises, promote the orderly development of the industry, and improve the overall level of the industry. |
| 2.3.2 | The NFSSF regulates the market and avoid unfair competition. | The standards of food labels and food additives in the NFSSF help standardize market orders and avoid unfair competition, such as misuse of food additions and false promotion activities. |
| 2.3.3 | The NFSSF is in line with the development direction of food & food-related industries and encourages innovation. | The establishment of the NFSSF leaves room for orderly developments of the industry and brings opportunities for the industry development, technological innovation, or other innovations. |
| 2.3.4 | The NFSSF helps control food safety risk at a reasonable cost. | The NFSSF and the requirements in the standards fully consider the industry cost, reduce the safety risk to an acceptable level, and avoid the pursuit of zero risk. |
| 2.4.1 | The NFSSF meets the specific social needs. | The lifecycle of the national food safety standards is public and transparent by adequate risk communication; the NFSSF considers and responds to social factors such as special industries and vulnerable population, and meets social needs. |
| 3.1.1 | The NFSSF is line with the laws and regulations, such as the Food Safety Law. | The establishment of the NFSSF complies with the requirements of relevant laws and regulations such as the Food Safety Law, the Agricultural Product Quality Safety Law, and the Anti-Food Waste Law. |
| 3.1.2 | The NFSSF is in line with the current national policies and development directions. | The NFSSF is in line with current national policies and development directions such as the “Opinions of the Central Committee of the Communist Party of China and the State Council on Deepening Reform and Strengthening Food Safety Work”, the “13th Five-Year National Food Safety Plan”, and the “14th Five-Year Plan for Circular Economy Development”, etc. |
| 3.2.1 | The NFSSF is closely connected with the edible agricultural product management system. | In the national food safety management system, the application and management scope of the NFSSF and the edible agricultural product management system are clear, and the two systems are closely integrated to ensure the safety and quality of food and edible agricultural products from production, processing to sales. |
| 3.2.2 | The NFSSF is complementary to the management of food safety by other relevant government departments. | The NFSSF is organically combined with the management of food safety by government departments such as environment, education, and market supervision; these different departments complement each other to jointly maintain food safety. |
| 3.2.3 | The NFSSF has a clear relationship with the food quality standard system. | The NFSSF guarantees food safety, and the food quality standard system guarantees the quality of food products. The relationship between the two systems is clear. |
| 3.3.1 | The NFSSF roughly matches with the Codex standard system and the food safety standard system of other developed countries in the world. | The NFSSF is basically in line with the Codex standard system and the regulations and standard systems of the major developed countries such as the United States, the European Union, Australia and New Zealand in terms of overall framework and design concept. |
